# Performance of ^18^ F-FAPI PET/CT in assessing glioblastoma before radiotherapy: a pilot study

**DOI:** 10.1186/s12880-022-00952-w

**Published:** 2022-12-24

**Authors:** Yutang Yao, Xiaofei Tan, Wenya Yin, Ying Kou, Xiaoxiong Wang, Xiao Jiang, Shirong Chen, Yongli Liu, Jun Dang, Jun Yin, Zhuzhong Cheng

**Affiliations:** 1 Department of Nuclear Medicine, Sichuan Cancer Hospital&Institute, Sichuan Cancer Center, University of Electronic Science and Technology of China, No.55, Section 4, South People’s Road, Sichuan 610041 Chengdu, China; 2 Department of Radiation Oncology, Sichuan Cancer Hospital&Institute, Sichuan Cancer Center, University of Electronic Science and Technology of China, No.55, Section 4, South People’s Road, 610041 Chengdu, China; 3 Institute of Isotope, China Institute of Atomic Energy, 102413 Beijing, China

**Keywords:** Glioblastoma, FAPI, PET/CT, Metabolic parameters, Molecular expression, MRI

## Abstract

**Background:**

We aimed to determine the performance of ^18^ F-FAPI PET/CT used for preprocedural assessment of glioblastoma before radiotherapy.

**Methods:**

Twelve glioblastoma patients having undergone incomplete surgical resection or biopsy were examined with ^18^ F-FAPI PET/CT and MRI scanning before radiotherapy. All patients had confirmed tumor residues according to findings of histopathological and/or long-term clinical and radiological follow-ups. Lesion characterization data, including SUV_max_ and tumor-to-background ratio (TBR) on PET/CT were attained. PET/CT and MRI findings were compared in terms of number of lesions. The correlation between immunohistochemistry, molecular expression, and PET/CT parameters was also evaluated.

**Results:**

^18^ F-FAPI PET/CT detected 16 FAPI-avid out of 23 lesions in 12 patients described on MRI. MRI was statistically different from ^18^ F-FAPI PET/CT for lesion detection according to the exact McNemar statistical test (*P* = 0.0156). The SUV_max_ and TBR of the glioblastomas was 7.08 ± 3.55 and 19.95 ± 13.22, respectively. The sensitivity and positive predictive value (PPV) of ^18^ F-FAPI PET were 69.6% and 100%, respectively. Neither the Ki-67 index nor the molecular expression was correlated with the FAPI-PET/CT parameters.

**Conclusion:**

^18^ F-FAPI PET/CT detects glioblastomas at a lower rate than MRI. However, the 100% PPV of the examination may make it useful for differentiating controversial lesions detected on MRI. The ^18^ F-FAPI-avid lesions are displayed more clearly probably due to a higher TBR. ^18^ F-FAPI PET/CT imaging might find application in glioblastoma biopsy and radiotherapy planning.

## Background

Glioblastoma is reported to be the most prevalent primary brain malignancy in adults, which represents one of the most rapidly growing glial tumors, accounting for about 57% of glioma. It is associated with a worse prognosis and a high recurrence rate[1]. Only some 6.8% of glioblastoma patients survive beyond five years [2]. As the most common non-invasive imaging technique to assess glioblastoma [3, 4], magnetic resonance imaging (MRI) has been employed as a diagnostic instrument for glioblastoma diagnosis, prognostication, and monitoring of therapy response [5–9]. Other methods, such as diffusion-weighted imaging (DWI), apparent diffusion coefficient (ADC), perfusion-weighted imaging (FWI), and magnetic resonance spectrum imaging (MRS) are also widely used in addition to conventional sequences [4, 6, 7, 10–13].

Positron emission tomography (PET) has been reported to provide additional information, which is helpful for identifying biological characteristics of tumor, differential diagnosis, and tumor size delineation and for an oncologist to plan a surgery, radiotherapy, and/or post-treatment tumor surveillance [14–17]. ^18^ F-fluorodeoxyglucose (^18^ F-FDG) is a commonly used PET tracer in clinical settings, which is known for diminished sensitivity due to increased physiologic uptake in the normal brain tissue [18]. When compared with glucose (^18^ F - FDG), amino acids including ^11^ C-MET, ^18^ F-FET, ^18^ F-FDOPA may be better tracers because they have higher specificity and lower signal/noise ratio [14, 17, 19].

Molecular-based PET-imaging that targets fibroblast activation protein (FAP) has been reported as a novel approach [20–24]. As a new diagnostic instrument, FAP-inhibitor (FAPI) PET imaging has found applications in a number of FAP-positive tumors, including glioblastoma [20, 25–29]. High tracer uptake in glioblastoma has been documented [28, 30, 31]. In this work, we performed ^18^ F-FAPI-PET imaging in 12 glioblastoma patients and attempted to evaluate its performance in pre-chemotherapy tumor assessment by evaluating uptake of FAP ligands in the tumor tissues.

## Materials and methods

### Patients

Patients with glioblastoma who underwent assessment before radiotherapy in our department between August 2020 and March 2022 were enrolled in this study. The diagnoses were confirmed by biopsy or incomplete surgical resection plus histological study before imaging and/or long-term clinical and radiological follow-up. The immunohistochemistry and molecular expression, such as Ki-67 proliferation index (Ki-67 index), O6-methylguanine-DNA methyltransferase (MGMT) promoter methylation status, Isocitrat Dehydrogenase (IDH) and telomerase reverse transcriptase (TERT) status, were collected. All patients were scanned with ^18^ F-FAPI PET/CT and MRI. The median time between the examinations was 3.5 days (range, 1 to 7 days).

### Radiopharmaceutical preparation

Radiosynthesis was as follows[32, 33]: ^18^ F-FAPI was automatically synthesized with modified AllinOne module (Trasis, Ans, Belgium). ^18^F-was attained on-site on a Sumitomo HM-10 cyclotron system (Sumitomo Heavy Industries, Tokyo, Japan), where [^18^O]H_2_O was irradiated with 10 MeV protons, and reacted with 0.15 mg NOTA-FAPI-04 (Paite Biotech, Beijing, China) (130 °C, 8 min, pH 4.0). An HLB cartridge (Waters Corporation) was used to collect the purified products. Quality of the final ^18^F-FAPI product was tested by HPLC (Shimadzu LC-15, Suzhou, China). Radiochemical purity was > 95%. Appearance, color, pH, and other quality controls were performed according to the current pharmacopoeias.

### PET/CT imaging

The patients were not specially prepped on the day of ^18^ F-FAPI PET/CT scanning. A Biograph mCT-64 scanner (Siemens, Germany) was used. The scanning was performed about 60 min after intravenous injection of ^18^ F-FAPI (0.12 millicurie/kg). The localizer was positioned with a scout head view. Low-dose CT (120 kV/110 mA) was then performed for anatomical localization and attenuation correction. Single-bed emission scans were obtained in 3-dimensional mode (acquisition time, 3 min). Reconstruction of data was done using an ordered subset expectation maximization iterative reconstruction algorithm (three iterations, 21 subsets). The emission data were corrected for random, scatter, and decay.

### MRI acquisition

MRI scans were acquired at 3 T. The sequences were: T1-weighted, T2-weighted, T2-weighted fluid-attenuated inversion recovery (FLAIR), T1-weighted gadoterate meglumine contrast-enhanced (CE-T1), MRS, ADC and DWI. Gadoterate meglumine (Gd-DOTA, DOTAREM, Guerbet, France) was intravenously administered in CE-T1 (pre-bolus dose, 0.1 mmol/kg).

### Image analysis

Two physicians with ten years of experience in brain tumor imaging evaluated the MRI images. Two nuclear medicine physicians who were experienced in PET/CT image assessment evaluated the PET/CT images. Disagreements were resolved by consensus. An abnormal focus of increased ^18^ F-FAPI uptake as compared with the background activity in the brain parenchyma was defined as positive on PET/CT. Standardized uptake values (SUV) were quantified using regions of interest (ROI). Should an area be found with increased uptake compared with the surrounding parenchyma, we drew a circular ROI over it where cystic or necrotic portions of the lesion was identified and avoided. Additionally, we drew an identical ROI over the contralateral normal cerebral cortex as a background. Tumor-to-background ratio (TBR) was calculated as the SUV_max_ of the lesion divided by the SUV_max_ of the contralateral normal cortex.The Response Assessment in Neuro-oncology (RANO) guidelines were used as the MRI reference. Lesions that were Gd-DOTA-enhancing, predominantly hyperintense with surrounding edema on the FLAIR sequence, or with a CHO/NAA ratio > 1.5 were considered positive on MRI images.

### Statistical analysis

We performed the statistical analyses using SPSS (version 22.0; IBM). Continuous variables are expressed as mean ± SD and categorical variables as numbers and percentages. The number of positive lesions was compared using exact McNemar statistical test. Inter-parameter correlations were calculated with the Spearman test. *P* < 0.05 was statistically significant.

## Results

### Patients’ characteristics

Twelve patients (aged 34–73 yrs, average 52 yrs) with glioblastomas confirmed by incomplete surgical resection or biopsy were enrolled in this study before radiotherapy. The characteristics are displayed in Table 1. The Ki-67 index of the glioblastomas was 42.92 ± 21.58%.

**Table 1:** Radiological and molecular characteristics of patients

Pt	Age, yr/Sex	Lesions (MRI)	Lesions (FAPI)	TBR	SUV_max_	Ki-67 index (%)	MGMT	TERT	IDH
1	48/M	4	1	16.76^#^	7.88	30	+	+	−
2	48/M	1	1	8.28^#^	7.52	60	+	−	−
3	59/M	1	1	19.21^#^	8.32	20	−	+	−
4	58/F	4	3	9.43^#^	7.99	40	−	+	−
				19.56	2.82	N/A	N/A	N/A	N/A
				22.03	3.48	N/A	N/A	N/A	N/A
5	34/F	1	1	8.88^#^	6.82	10	+	−	+
6	56/F	4	1	26.59^#^	8.64	50	−	+	−
7	48/F	2	2	10.98^#^	7.20	80	−	+	−
				21.05	1.94	N/A	N/A	N/A	N/A
8	36/F	1	1	61.69^#^	8.27	60	+	−	−
9	73/M	1	1	27.38^#^	8.63	20	+	+	−
10	58/M	1	1	13.06^#^	7.50	70	+	−	−
11	49/F	2	2	7.90^#^	3.41	35	+	+	−
				16.19	5.49	N/A	N/A	N/A	N/A
12	55/M	1	1	30.21^#^	17.34	40	+	−	−
Total		23	16	19.95 ± 13.22	7.08 ± 3.55				

Pt, patient; MGMT: MGMT promoter methylation; TERT: TERT mutation; IDH:IDH mutation; #: specimen undergoing biopsy or surgical resection for immunohistochemistry, and molecular expression

According to the molecular expression, MGMT promoter methylation was found in 7 patients (58.3%), TERT mutation in 8 patients (66.7%), and IDH mutation only in 1 patient (8.3%).

### MRI image and ^18^ F-FAPI PET/CT characteristics

**Fig. 1:**
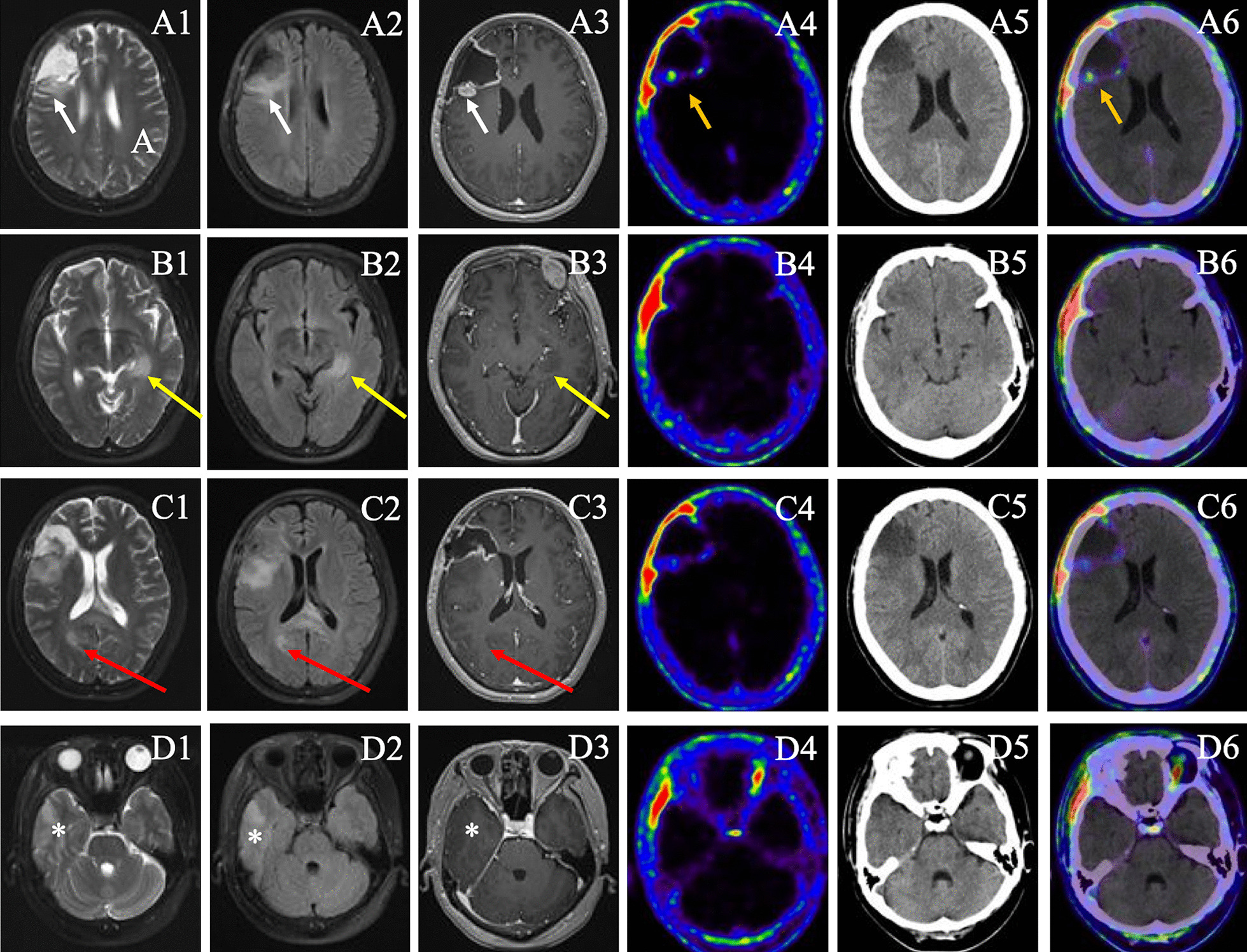
The ^18^ F-FAPI PET/CT and MRI (T2, FLAIR, CE-T1) images of patient 6.** A1**–**D1** were images of T2 sequence,** A2**–**D2** were images of FLAIR,** A3**–**D3** were images of enhanced T1,** A4**–**D4** were images of ^18^ F-FAPI PET,** A5**–**D5** were images of non-enhanced CT,** A6**–**D6** were images of fusion of ^18^ F-FAPI PET and CT. The patient underwent incomplete surgical resection of the glioblastoma in the right frontal lobe. Four lesions were detected on MRI (**A1**-**D1**, short arrow, yellow arrow, red arrow, and asterisk). Only one ^18^ F-FAPI-avid lesion was observed on PET/CT (orange arrow, **A4**-**A6**).

**Fig. 2:**
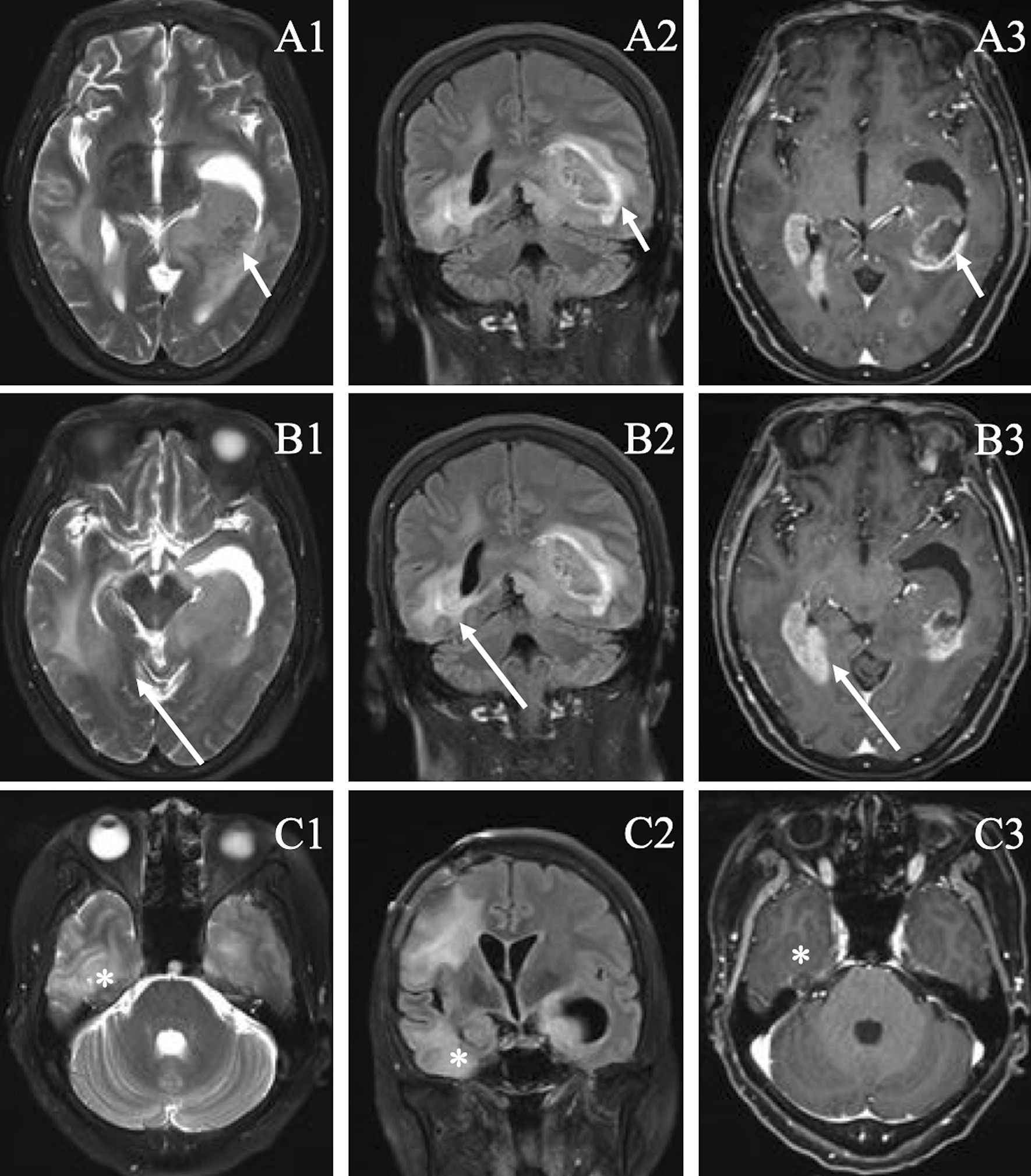
The non-FAPI-avid lesions were confirmed by follow-up MRI (T2, FLAIR, CE-T1) 6 months later.** A1**–**C1** were images of T2 sequence,** A2**–**C2** were images of FLAIR, A3-C3 were images of enhanced T1. All enlarged and partially mixed signals were heterogeneously enhanced (short arrow, long arrow, and asterisk), indicating disease progression

MRI detected 23 lesions in 12 patients, whereas ^18^ F-FAPI PET/CT detected 16 FAPI-avid lesions (Table 1; Figs. 1 and 2). Performance of MRI and ^18^ F-FAPI PET/CT was significantly different in terms of lesion detection (*P* = 0.0156). The SUV_max_ and TBR were 7.08 ± 3.55 and 19.95 ± 13.22 in the FAPI-avid lesions, respectively (Table 2).

**Table 2:** Correlations between Ki-67 index, molecular expression, and FAPI-PET/CT parameters

	TBR	SUV_max_
Ki-67	r = 0.018, *P* = 0.957	r = − 0.095, *P* = 0.769
MGMT status	r = 0.269, *P* = 0.397	r = 0.073, *P* = 0.821
TERT status	r = − 0.073, *P* = 0.821	r = 0.073, *P* = 0.821
IDH status	r = − 0.306, *P* = 0.334	r = − 0.393, *P* = 0.206

The lesions were subsequently validated with biopsy (3, 13.0%), biopsy after complete or incomplete resection (10, 43.5%), and/or radiological follow-up (10, 43.5%). The sensitivity and positive predictive value (PPV) of ^18^ F-FAPI PET/CT were 69.6% and 100%, respectively.

Neither the Ki-67 index nor the molecular expression was correlated with the ^18^ F-FAPI-PET/CT parameters. (*P* > 0.05, Table 2)

## Discussion

FAP overexpression and cancer associated fibroblasts (CAFs) found in tumor stroma are believed to cause the FAP-positive signaling in extracranial tumors [21, 34]. Imaging techniques that target FAP are novel promising instruments for visualizing tumor stroma. Excellent performance of FAPI PET/CT has been reported in various tumors including glioblastoma [21, 28].

Although fibroblasts do not exist in the brain, FAP-positive vessels in glial tumors, FAP-positive foci of neoplastic cells in gliomas, as well as FAP- positive stromal cells that function similarly to CAFs in epithelial cancers have been reported by several researchers[35–38]. FAP and FAP mRNA are overexpressed in most glioblastomas, especially in the mesenchymal subtype [37]. Some 65% of glioblastomas are FAP-positive intraparenchymally. In our pilot study, ^18^ F-FAPI PET/CT detected 69.6% (16/23) of all lesions, which could be attributed to the varying FAP expressions in different molecular glioblastoma subtypes [37] or the status of the blood-brain barrier [19]. Although its sensitivity was not high, the PPV of ^18^ F-FAPI PET/CT was up to 100%, which may be useful for differentiating controversial lesions detected on MRI. However, prospective studies based on larger samples are still needed to validate the findings.

To this day MRI remains the most used imaging modality for glioblastoma diagnosis and evaluation of treatment response and prognosis [1, 4, 10]. A number of advanced MRI techniques such as functional MRI (fMRI), diffusion tensor imaging (DTI), and MRS have been adopted for tumor treatment including preoperative surgical planning or as an instrument to distinguish post-operative vascular damage from residual enhancing tumor[9, 12, 13, 39]. However, it is highly challenging to assess sub-regions of tumors in MRI scans visually because of their anatomic complexity. This can be even more true in post-operative settings[13]. It is a time-consuming task for clinicians to delineate the targets and critical structures, where observer biases are probable. Up to 20% intra- and 28% inter-rater variability have been reported for determining glioma boundaries[40]. As a result, boundary delineation of radiotherapy target volume could be inaccurate. Advanced molecular imaging could be helpful at this point[41]. Although the lesion detection rate was lower on ^18^ F-FAPI PET/CT than on MRI in our study, the TBR of the lesion was high (range 7.90-30.21) and the boundaries were better visualized due to the low FAPI distribution in the normal cerebral tissues. FAPI PET/CT shows potential for application in biopsy and radiotherapy planning for glioblastoma [28, 30, 42]. According to a Polish study, target volume delineation based on MRI and FAPI PET is differentiated[30]. Gross tumor volumes (GTVs) delineated using FAPI PET plus MRI is significantly higher compared with MRI alone[30]. Unfortunately, the metabolic GTVs were not calculated in this study due to the threshold value of MTV in FAPI PET/CT was still indeterminate. The further study is needed to analyze the different threshold value of MTV in FAPI PET/CT compared with the GTVs on MRI or RT planning.

Ki-67 index is a quantitative measure of cell proliferation in histopathological assessment of glioblastoma and many other tumors [43]. Ki-67 index and TERT or IDH mutation are positively associated with overall survival of glioblastoma patients and a lower Ki-67 index and IDH-wildtype are linked with poor prognosis[43, 44]. However, our findings failed to establish any correlations between the Ki-67 index or the molecular expression with the FAPI-PET/CT parameters, such as MGMT promoter methylation, TERT, and IDH mutation. Yet, only one of the glioblastoma patients in our study had IDH mutation. A larger sample may be needed in future research to further investigate our finding.

### Limitation

Our sample size was limited with 12 patients and 23 lesions. As a pilot study, our findings are preliminary and should be interpreted with caution. Another limitation to our study was that some of the lesions were not labeled with FAP on immunohistochemistry. This prevented us from evaluating the correlation between FAP expression and the PET/CT parameters. Further studies with larger samples are needed to validate our results, especially the correlation findings.

## Conclusion

Despite a lower lesion detection rate than MRI, the PPV of ^18^ F-FAPI PET/CT was 100%, suggesting it as a useful examination for indeterminate lesions detected on MRI. ^18^ F-FAPI PET/CT visualizes lesions more clearly with a high TBR, possibly because of lower FAPI distribution in the normal cerebral tissues. The technique may find applications in target volume delineation in radiotherapy of glioblastoma.

## Data Availability

The raw data supporting the conclusions of this article will be made available by the authors, without undue reservation.
